# A phase 3 randomized controlled trial of a COVID-19 recombinant vaccine S-268019-b versus ChAdOx1 nCoV-19 in Japanese adults

**DOI:** 10.1038/s41598-024-57308-3

**Published:** 2024-04-29

**Authors:** Satoshi Iwata, Andrew J. Pollard, Yukio Tada, Shinya Omoto, Risa Y. Shibata, Kenji Igarashi, Takahiro Hasegawa, Mari Ariyasu, Takuhiro Sonoyama

**Affiliations:** 1 Department of Infectious Diseases, National Cancer Center Hospital, Tokyo, Japan; 2 Oxford Vaccine Group, Department of Paediatrics, University of Oxford, Oxford, UK; 3 the NIHR Oxford Biomedical Research Centre, Oxford, UK; 4 Drug Development and Regulatory Science Division, Shionogi & Co., Ltd., Osaka, Japan; 5 Biopharmaceutical Research Division, Shionogi & Co., Ltd., Osaka, Japan

**Keywords:** Immunogenetics, Vaccines

## Abstract

We assessed S-268019-b, a recombinant spike protein vaccine with a squalene-based adjuvant, for superiority in its immunogenicity over ChAdOx1 nCoV-19 vaccine among adults in Japan. In this multicenter, randomized, observer-blinded, phase 3 study, severe acute respiratory syndrome coronavirus 2 (SARS-CoV-2)–naïve participants (aged ≥ 18 years, without prior infection or vaccination against SARS-CoV-2) were randomized (1:1) to receive either S-268019-b or ChAdOx1 nCoV-19 as two intramuscular injections given 28 days apart. Participants who provided consent for a booster administration received S-268019-b at Day 211. The primary endpoint was SARS-CoV-2 neutralizing antibody (NAb) titer on Day 57; the key secondary endpoint was the seroconversion rate for SARS-CoV-2 NAb titer on Day 57. Other endpoints included anti–SARS-CoV-2 S-protein immunoglobulin (Ig)G antibody titer and safety. The demographic and baseline characteristics were generally comparable between S-268019-b (n = 611) and ChAdOx1 nCoV-19 (n = 610) groups. S-268019-b showed superior immunogenicity over ChAdOx1 nCoV-19, based on their geometric mean titers (GMTs) and GMT ratios of SARS-CoV-2 NAb on Day 57 by cytopathic effect assay (GMT [95% confidence interval {CI}] 19.92 [18.68, 21.23] versus 3.63 [3.41, 3.87]; GMT ratio [95% CI] 5.48 [5.01, 6.00], respectively; two-sided *p*-values < 0.0001). Additionally, NAb measured using a cell viability assay also showed similar results (GMT [95% CI] 183.25 [168.04, 199.84] versus 24.79 [22.77, 27.00]; GMT ratio [95% CI] 7.39 [6.55, 8.35] for S-268019-b versus ChAdOx1 nCoV-19, respectively; *p* < 0.0001). The GMT of anti–SARS-CoV-2 S-protein IgG antibody was 370.05 for S-268019-b versus 77.92 for ChAdOx1 nCoV-19 on Day 57 (GMT ratio [95% CI] 4.75 [4.34, 5.20]). Notably, immune responses were durable through the end of the study. S-268019-b elicited T-helper 1 skewed T-cell response, comparable to that of ChAdOx1 nCoV-19. After the first dose, the incidence of solicited systemic treatment-related adverse events (TRAEs) was higher in the ChAdOx1 nCoV-19 group, but after the second dose, the incidence was higher in the S-268019-b group. Headache, fatigue, and myalgia were the most commonly reported solicited systemic TRAEs, while pain at the injection site was the most frequently reported solicited local TRAE following both doses in both groups. No serious treatment-related adverse serious TRAEs events were reported in the two groups. S-268019-b was more immunogenic than ChAdOx1 nCoV-19 vaccine and was well tolerated (jRCT2051210151).

## Introduction

Coronavirus disease 2019 (COVID-19), caused by severe acute respiratory syndrome coronavirus 2 (SARS-CoV-2), has caused over 765 million confirmed cases and 7.7 million reported deaths worldwide as of December 12, 2023^1^. Since the emergence of COVID-19 in 2020, many vaccines have been developed and a few approved for emergency use by the World Health Organization after they met rigorous, scientific standards for safety, short-term effectiveness, and the manufacturing quality mandated for such expedited authorization. Multiple vaccines with diverse platforms have been developed against SARS-CoV-2 infection in the past 3 years: 50 vaccines are approved in one or more nations and 183 new vaccine candidates are in the clinical development pipeline globally as of March 2023^2^. Although a majority of the world’s population is vaccinated, the risk of waning immunity following natural infection or immunization and the risk of new emerging variants of concern^3,4^ make booster administration of vaccines increasingly relevant to protect the vulnerable populations.

Seven vaccines have been approved to date in Japan: (1) BNT162b2 and (2) mRNA-1273, both mRNA vaccines; (3) ChAdOx1 nCoV-19, a viral vector vaccine containing a modified, replication-deficient chimpanzee adenovirus ChAdOx1; (4) NVX-CoV2373, a recombinant nanoparticle vaccine; (5) Ad26.COV2.S, a recombinant, replication-incompetent human adenovirus type 26 vector, (6) DS-5670, an Omicron XBB.1.5-adapted monovalent mRNA vaccine, and (7) KD-414, an inactivated and self-amplifying mRNA vaccine. These vaccines have been highly effective compared with placebo in their pivotal clinical studies^5–9^. Conducting placebo-controlled disease endpoint efficacy trials for new vaccine candidates is challenging, as only a few individuals globally are still unvaccinated and uninfected. As neutralizing antibodies are considered a surrogate marker of the conferred protection against SARS-CoV-2 infection^10–12^, appropriately designed active comparator-controlled, immuno-bridging studies utilizing neutralizing antibody titers as a suitable primary endpoint to predict vaccine effectiveness are an acceptable alternative approach for authorizing new vaccines^13,14^.

Shionogi & Co., Ltd., has been developing the S-268019-b vaccine, comprising a full-length recombinant SARS-CoV-2 S-protein, modifying putative furin cleavage site at the middle of the protein and two proline substitutions in the C-terminal region to secure stability, produced using the baculovirus expression system in rhabdovirus-free insect cells, with a squalene-based adjuvant (A-910823) in an oil-in-water emulsion formulation^15^. We have previously reported that the S-268019-b vaccine elicited robust immunogenic response 4 weeks after the second dose of the vaccine in healthy Japanese adult participants in an interim analysis of the double-blinded phase 1/2 trial^16^. Moreover, S-268019-b was well tolerated and immunogenic in a large participant pool comprising SARS-CoV-2–naïve adults and elderly participants, and adult participants with a history of either prior infection or vaccination against SARS-CoV-2 for 4 weeks following the second injection in an open-label study in Japan^16^.

Based on the encouraging interim results from the phase 1/2 study followed by the phase 2/3 study, this phase 3 study reported here was planned. Given the availability of multiple approved vaccines in Japan by the end of 2021, we decided to use the ChAdOx1 nCoV-19 vaccine as the control. We aimed to evaluate whether the immunogenicity of S-268019-b (10 μg antigen strength used based on the phase 1/2 study results^16^) is superior to that of ChAdOx1 nCoV-19 after two intramuscular injections in SARS-CoV-2–naïve participants. Herein, we report the results from an analysis of the phase 3 study, with safety and immunogenicity data collected until 4 weeks following the second dose of S-268019-b and ChAdOx1 nCoV-19 vaccines and immunogenicity data collected until the end of the study (393 days) following initial vaccination.

## Methods

### Study design and participants

This multicenter, randomized, observer-blind, active-controlled study included participants aged ≥ 18 years without a history of a laboratory-confirmed diagnosis of SARS-CoV-2 infection or COVID-19, or previous vaccination against SARS-CoV-2 in Japan from January 17, 2022, to January 30, 2022 (jRCT2051210151, the first registration date: January 13, 2022). Detailed inclusion and exclusion criteria are described in Supplementary Fig. 1.

### Randomization and masking

Over 1000 eligible participants were randomized 1:1 and allocated to either the S-268019-b group or the active control group on Day 1 by using a Web response interactive system. The assignment was stratified by age groups (18–39 years, 40–64 years, and ≥ 65 years). This was an observer-blinded study; the participants, investigators, and site staff not involved in preparation and vaccination were blinded to the study intervention. Since S-268019-b and the active control vaccine were distinguishable by appearance, a pharmacist preparing study interventions and a vaccine administrator were unblinded and were not allowed to communicate with the participants, investigators, and other site staff about participants’ treatment assignment. While assignment of participants to the intervention groups was unblinded at the time of the primary immunogenicity analysis and the database lock, participants remained blinded until the end of the study. The Web response interactive system will be programmed with blind-breaking instructions. In case of an emergency or participant safety concerns, the investigator had the sole responsibility for determining if unblinding of a participant’s intervention assignment or stoppage was warranted.

### Study intervention

The study comprised a screening period (Day − 28 to Day 1 pre-dose), an evaluation period (Day 1 post-dose to Day 57), and a follow-up period (Day 58 to Day 393; Supplementary Fig. 2). Participants were administered the S-268019-b (10 μg antigen with 0.25 mL A-910823 adjuvant in 0.5 mL oil-in-water emulsion) or ChAdOx1 nCoV-19 suspension injection, both given intramuscularly at a 4-week interval (first dose: Day 1, second dose: Day 29). Participants visited the study site on Days 1, 29, 43, 57, 97, 211, and 393 for administration of intervention or further assessments. Participants who had given additional consent to cellular immunity and Th1/Th2 balance analyses also visited the study site on Day 15 for the scheduled visit. Additionally, participants who provided consent for a booster dose were administered S-268019-b on Day 211 regardless of the vaccine received on Days 1 and 28.

### Outcomes

**Figure 1:**
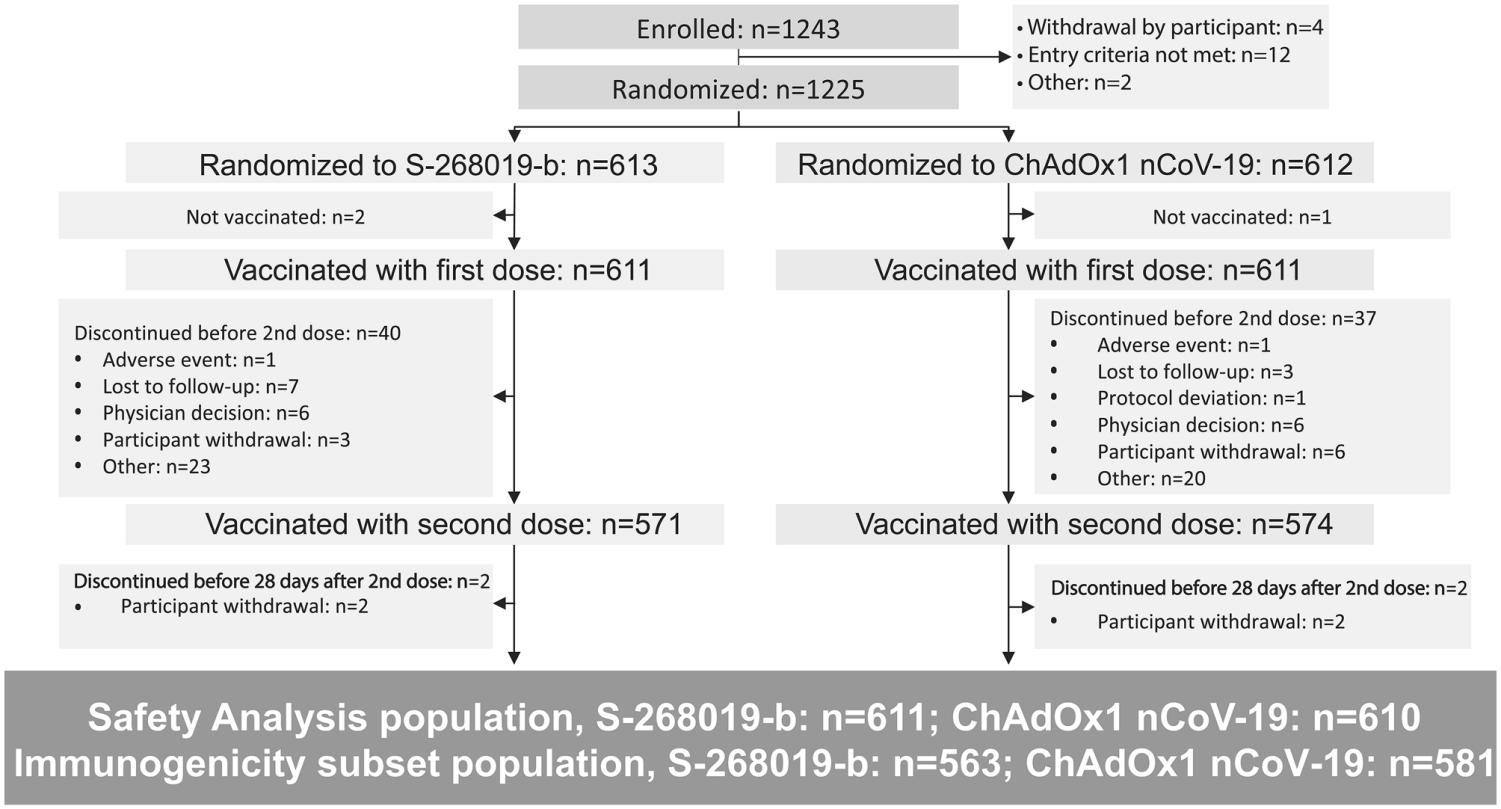
Participant disposition. Safety analysis population included randomized participants who received at least one dose of study intervention. Immunogenicity subset population included participants selected from all randomized participants who were included in the mITT population and had at least 1 valid immunogenicity result after the first dose of vaccination. mITT, modified intent-to-treat. One participant who received ChAdOx1 nCoV-19 was excluded due to double enrolment; therefore, the safety analysis population for ChAdOx1 nCoV-19 included 610 participants.

The primary endpoint was SARS-CoV-2 neutralizing antibody titer 28 days following the second vaccination (i.e., Day 57 of the study) (Fig. 1).

The key secondary endpoint was seroconversion rate for SARS-CoV-2 neutralizing antibody titer at 28 days after the second dose. Seroconversion rate was defined as the percentage of participants with a ≥ fourfold increase in the post-vaccination antibody titer from baseline. If the post-vaccination antibody titer was below the lower level of quantification (LLoQ), participants were considered seroconversion negative. Other secondary endpoints included safety (number of specific adverse events [AEs], serious AEs [SAEs], etc.), efficacy (COVID-19 incidence, asymptomatic COVID-19 incidence, etc.), and immunogenicity parameters (anti–SARS-CoV-2 S-protein IgG antibody titer, neutralizing antibody titer, etc.). Safety was assessed by monitoring solicited local and systemic AEs, unsolicited AEs, treatment-related AEs (TRAEs), SAEs, medically attended adverse events (MAAEs), and AEs of special interest (AESIs). Solicited local and systemic AEs were recorded for 7 days following each vaccination using an e-diary. Unsolicited non-serious AEs were collected from the date of signing of the informed consent form to Day 57. SAEs, MAAE, and AESIs were collected until Day 393.

Exploratory endpoints included neutralizing antibody titer against variants of interest and variants of concern, and cellular immunity assessed using cytokine-producing cell count by interferon-gamma (IFN-γ) ELISPOT assay and Th1/Th2 balance by intracellular cytokine staining assay. The methodology for quantification of the anti-spike protein IgG titer, neutralizing antibody titer, IFN-γ ELISPOT assay, and intracellular cytokine staining by flow cytometry (ICS-FCM) has been previously described^16^. Neutralizing antibody levels for the SARS-CoV-2 variants were assessed using the ancestral strain (D614E) as described in the phase 2/3 booster publication^17^. Notably, the values below LLoQ were replaced by values of 0.5 × LLoQ. Additional methods are described in Supplementary Appendix.

### Ethics approval and statement

The study was approved by Review Board of Human Rights and Ethics for Clinical Studies Institutional Review Board in Clinical Research Hospital Tokyo, Shinei Diabetes Clinic, Yamazaki Otorhinolaryngology and Vertigo Clinic, Public Health Insurance Association Clinic, Shinagawa Strings Clinic, Tokyo Asbo Clinic, Motomachi Takatsuka Naika Clinic, Dojinkinenkai Meiwa Hospital, Shimamura Memorial Hospital, Koseikai Yotsubashi Clinic, and Tenjin Sogo Clinic, by Takahashi Pediatric Clinic Institutional Review Board in Irie Medical Clinic, Aijinkai Takatsuki General Hospital, and Nihonbashi Sakura Clinic, by Tokyo-Eki Center-building Clinic Institutional Review Board in Tokyo-Eki Center-Building Clinic and Fukuwa Clinic, by Institutional Review Board, P1 Clinic, Keiko-kai Medical Corporation in Higashi Shinjuku Clinic, by Yoyogi Mental Clinic Institutional Review Board in Samoncho Clinic, by Medical Corporation Cattleyakai Dr.Mano Medical Clinic Institutional Review Board in Sapporo Odori Endoscopy Clinic, and by Sekino Hospital Institutional Review Board in Sekino Hospital (Supplementary Table 1). The study was conducted in accordance with the protocol, the Declaration of Helsinki and Council for International Organizations of Medical Sciences (CIOMS) International Ethical Guidelines, the International Council for Harmonisation of Technical Requirements for Pharmaceuticals for Human Use (ICH) Good Clinical Practice Guidelines, and other applicable laws and regulations. All participants provided written informed consent.

### Statistical analyses

Sample size determination indicated that a total of 882 participants from the immunogenicity subset whose SARS-CoV-2 neutralizing antibody titers were observed at 28 days after the second dose were required to demonstrate the superiority with at least 90% power at a two-sided significance level of 0.05, assuming the standard deviation (SD) of the natural log-transformed titers of SARS-CoV-2 neutralizing antibody titers to be 1.2 and the true geometric mean titer ratio (GMTR) between S-268019-b and the ChAdOx1 nCoV-19 vaccine to be 1.3. Considering early termination, the target number of participants who had immunogenicity assessment and had no SARS-CoV-2 seropositivity at baseline was 1,000.

The following analysis sets were included (1) full analysis set (FAS) containing all randomized participants who received at least one dose of the study intervention; (2) modified intent-to-treat (mITT) population comprising participants in the FAS but excluding participants who had evidence of past or present SARS-CoV-2 infection at baseline; (3) immunogenicity subset containing participants selected from all randomized participants who were included in the mITT population and had at least one valid immunogenicity result after the first vaccine dose; and (4) safety analysis set comprising randomized participants who received at least one dose of the study intervention.

The immunogenicity subset population was used for the following hypothesis testing:

Null hypothesis: $${\mu }_{\text{A}}$$=$${\mu }_{\text{c}}$$

Alternative hypothesis: ${\mu }_{\text{A}}\ne {\mu }_{\text{c}}$where ${\mu }_{\text{A}}$ and ${\mu }_{\text{C}}$ are the geometric means of SARS-CoV-2 neutralizing antibody titers at 28 days following the second vaccination for S-268019-b recipients and that for ChAdOx1 nCoV-19 recipients, respectively. If the lower limit of the 95% confidence interval (CI) for the GMTR (S-268019-b to the ChAdOx1 nCoV-19 vaccine) was greater than 1, superiority of S-268019-b was confirmed.

For the primary endpoint analysis, superiority assessment was planned assuming a low proportion of neutralizing antibody titer below the LLoQ. Otherwise, post hoc analysis by Wilcoxon rank-sum test, which is a non-parametric statistical hypothesis test, was planned to compare the two groups. No statistical analysis was planned for neutralizing antibody responses against virus variants.

## Results

Overall, 1,243 participants were enrolled, of which 1,225 were randomized to receive S-268019-b (n = 613) or ChAdOx1 nCoV-19 (n = 612; Fig. 1). Of the 18 participants who were excluded from the study after enrollment, the most common reason was that participants no longer met the inclusion criteria (n = 12). Among the randomized participants, 2 and 40 participants in the S-268019-b group and 1 and 37 in the ChAdOx1 nCoV-19 group discontinued the study before and after the first dose, respectively. Consequently, 571 and 574 participants received second doses of S-268019-b and ChAdOx1 nCoV-19 vaccines, respectively. Although immunogenicity subset population included 563 and 581 participants in the S-268019-b and ChAdOx1 nCoV-19 groups, only 497 and 514 participants were eligible for immunogenicity analysis at Day 57 as measurements obtained after a postbaseline dose of approved vaccine, a postbaseline positive anti-N-protein antibody or SARS-CoV-2 RT-PCR test result, or a postbaseline onset of COVID-19 were not included in the analysis.

Baseline demographic characteristics of the participants in the S-268019-b and ChAdOx1 nCoV-19 groups were similar and are described in Table 1. Less than a quarter of the participants in both S-268019-b and ChAdOx1 nCoV-19 groups had a history of smoking (23.4% and 23.0%, respectively), while hypertension was the most common risk factor in both groups (6.9% and 9.3%, respectively).

**Table 1:** Baseline demographics of participants from the safety analysis set.

Characteristic	S-268019-b	ChAdOx1 nCoV-19
	(N = 611)	(N = 610)
Age (years), mean (SD)	43.6 (12.8)	44.0 (12.7)
Age group (years)		
≥ 18 to < 40	219 (35.8)	219 (35.9)
≥ 40 to < 65	366 (59.9)	365 (59.8)
≥ 65	26 (4.3)	26 (4.3)
Age group (years)		
< 30	106 (17.3)	107 (17.5)
≥ 30 to < 40	113 (18.5)	112 (18.4)
≥ 40 to < 50	176 (28.8)	160 (26.2)
≥ 50 to < 60	157 (25.7)	166 (27.2)
≥ 60 to < 65	33 (5.4)	39 (6.4)
≥ 65	26 (4.3)	26 (4.3)
BMI group (kg/m^2^)		
< 30	559 (91.5)	557 (91.3)
≥ 30	52 (8.5)	53 (8.7)
Sex		
Male	405 (66.3)	397 (65.1)
Female	206 (33.7)	213 (34.9)
Childbearing potential (for women), Yes	158 (76.7)	148 (69.5)
Body mass index (kg/m^2^), mean (SD)	23.66 (4.36)	24.02 (4.39)
Baseline results of anti–SARS-CoV-2 N-protein antibody test		
Positive	32 (5.3)	17 (2.8)
Negative	577 (94.7)	593 (97.2)
Race		
American Indian or Alaska Native	0	1 (0.2)
Asian	611 (100.0)	609 (99.8)
Smoking status, yes	143 (23.4)	140 (23.0)
Risk factors present		
No risk factors	544 (89.0)	527 (86.4)
Diabetes	11 (1.8)	17 (2.8)
Serious heart disease	2 (0.3)	2 (0.3)
Chronic airway and lung disease	7 (1.1)	14 (2.3)
Chronic liver disease	9 (1.5)	6 (1.0)
Hypertension or high blood pressure	42 (6.9)	57 (9.3)
Hematological disorders	1 (0.2)	3 (0.5)
Cerebrovascular disease affecting blood vessels and blood supply to the brain	3 (0.5)	1 (0.2)
Cancer	0	1 (0.2)
Chronic kidney disease	1 (0.2)	1 (0.2)
Neurologic conditions	0	1 (0.2)

*BMI* Body mass index, *SARS-CoV-2* Severe acute respiratory syndrome coronavirus 2, *SD* Standard deviation.

Data are presented as n (%) unless specified otherwise.

In total, 609 and 610 participants from the S-268019-b and ChAdOx1 nCoV-19 groups underwent an anti–SARS-CoV-2 N-protein antibody test.

**Figure 2:**
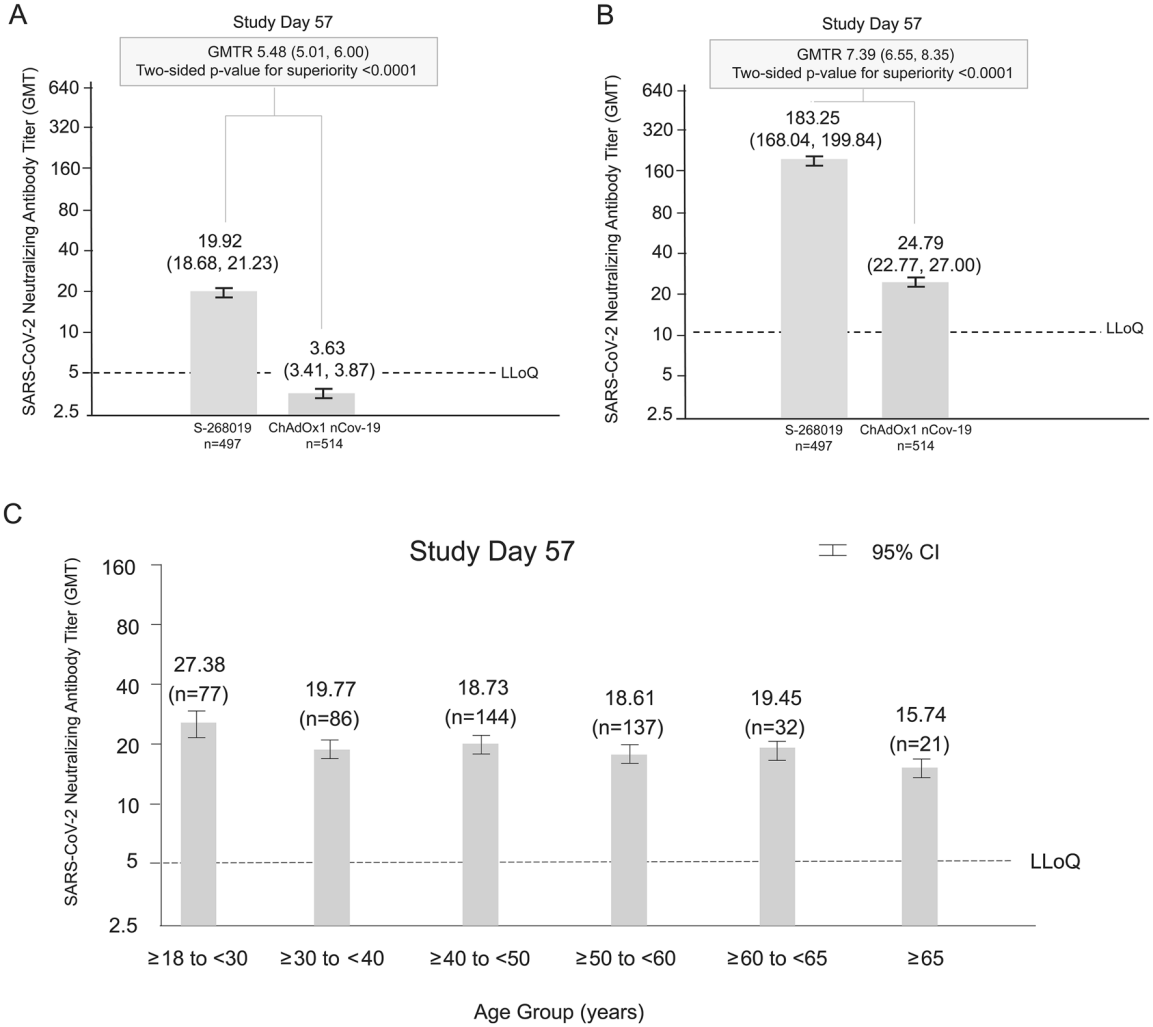
GMTs for SARS-CoV-2 neutralizing antibody: immunogenicity subset using two different methods: (**a**) cytopathic effect assay; (**b**) cell viability assay; and (**c**) GMTs for SARS-CoV-2 neutralizing antibody measured using the cytopathic effect assay in the S-268019-b group: immunogenicity subset in subgroups by age. CI, confidence interval; GMT, geometric mean titer; GMTR, geometric mean titer ratio; LLoQ, lower limit of quantification; SARS-CoV-2, severe acute respiratory syndrome coronavirus 2. Wilcoxon rank-sum test was applied as a post hoc analysis for both methods because there was a certain level of proportion of neutralizing antibody titer below the LLoQ in the ChAdOx1 nCoV-19 group in the results of both methods.

The two-dose regimen of intramuscular injection of S-268019-b was more immunogenic than that of ChAdOx1 nCoV-19, based on the SARS-CoV-2 neutralizing antibody titer measured by cytopathic effect assay 28 days after the second dose of the respective vaccine (geometric mean titer [GMT] 19.92 vs. 3.63; GMTR [95% CI] 5.48 [5.01, 6.00]; two-sided *p*-value for superiority < 0.0001; Fig. 2a). Robust immunogenicity of S-268019-b was evident across all age groups (Fig. 2c). Notably, the difference in the initial immune responses seen at Day 57 between both vaccines were maintained following administration of a booster dose on Day 211 (Supplementary Fig. 3). Indeed, GMTs on Days 239, 302, and 393 were substantially higher than those seen on Day 211 (considered baseline for the booster dose). Additionally, GMTs for the neutralizing antibody titers continued to be substantially higher in the S-268019-b vs. the ChAdOx1 nCoV-19 group. The seroconversion rate in participants receiving S-268019-b for SARS-CoV-2 neutralizing antibody using the cytopathic effect assay 28 days after the second dose of the vaccine was noninferior to the response in the ChAdOx1 nCoV-19 group (seroconversion rate [95% CI] 91.1% [88.3%, 93.5%] vs. 8.2% [6.0%, 10.9%]; one-sided *p*-value for non-inferiority < 0.0001; Supplementary Fig. 4). Since approximately 60% of the neutralizing antibody titers in the ChAdOx1 nCoV-19 group were below the LLoQ (Supplementary Fig. 5a), to confirm the robustness of the result, an additional neutralizing antibody measurement by a cell viability assay, which is more sensitive than the cytopathic effect assay, was conducted. The cell viability assay also showed similar results (GMT [95% CI] 183.25 [168.04, 199.84] vs. 24.79 [22.77, 27.00]; GMT ratio [95% CI] 7.39 [6.55, 8.35], *p* < 0.0001; Fig. 2b). Moreover, the neutralizing antibody titers were statistically significantly different between groups when assessed using the Wilcoxon rank-sum test, a post hoc analysis for both methods (both *p* < 0.0001). GMT in the S-268019-b group was comparable to that observed in a set of convalescent serum samples from symptomatic outpatients who had recovered from COVID-19 (GMT in the convalescent serum samples was 145.9 [n = 30]). The seroconversion rate following S-268019-b for SARS-CoV-2 neutralizing antibody measured by cell viability assay 28 days after the second dose of the vaccine was higher than that in the ChAdOx1 nCoV-19 group (seroconversion rate [95% CI] 98.8% [97.4%, 99.6%] vs. 66.6% [61.7%, 70.0%]; data not shown). Approximately 20% of the neutralizing antibody titers in the ChAdOx1 nCoV-19 group were below LLoQ (Supplementary Fig. 5b). The GMT of anti–SARS-CoV-2 S-protein IgG antibody titer was higher in the S-268019-b group than the ChAdOx1 nCoV-19 group 28 days after the second dose (GMT 370.05 vs. 77.92; GMTR [95% CI], 4.75 [4.34, 5.20]; Supplementary Fig. 6). Similar to the trend seen for neutralizing antibody titer, anti–SARS-CoV-2 S-protein IgG responses were durable. GMTs for anti–SARS-CoV-2 S-protein IgG titers were substantially higher through Day 393 for both groups when compared to the baseline. Moreover, anti–SARS-CoV-2 S-protein IgG responses in the S-268019-b group continued to be substantially higher than those in the ChAdOx1 nCoV-19 group after Day 57 through Day 393 (Supplementary Fig. 7). The seroconversion rate (95% CI) after S-268019-b for anti–SARS-CoV-2 S-protein IgG antibody 28 days after the second dose was comparable with that in the ChAdOx1 nCoV-19 group (99.8% [98.9%, 100.0%] vs. 98.2% [96.7% vs. 99.2%]; data not shown).

**Figure 3:**
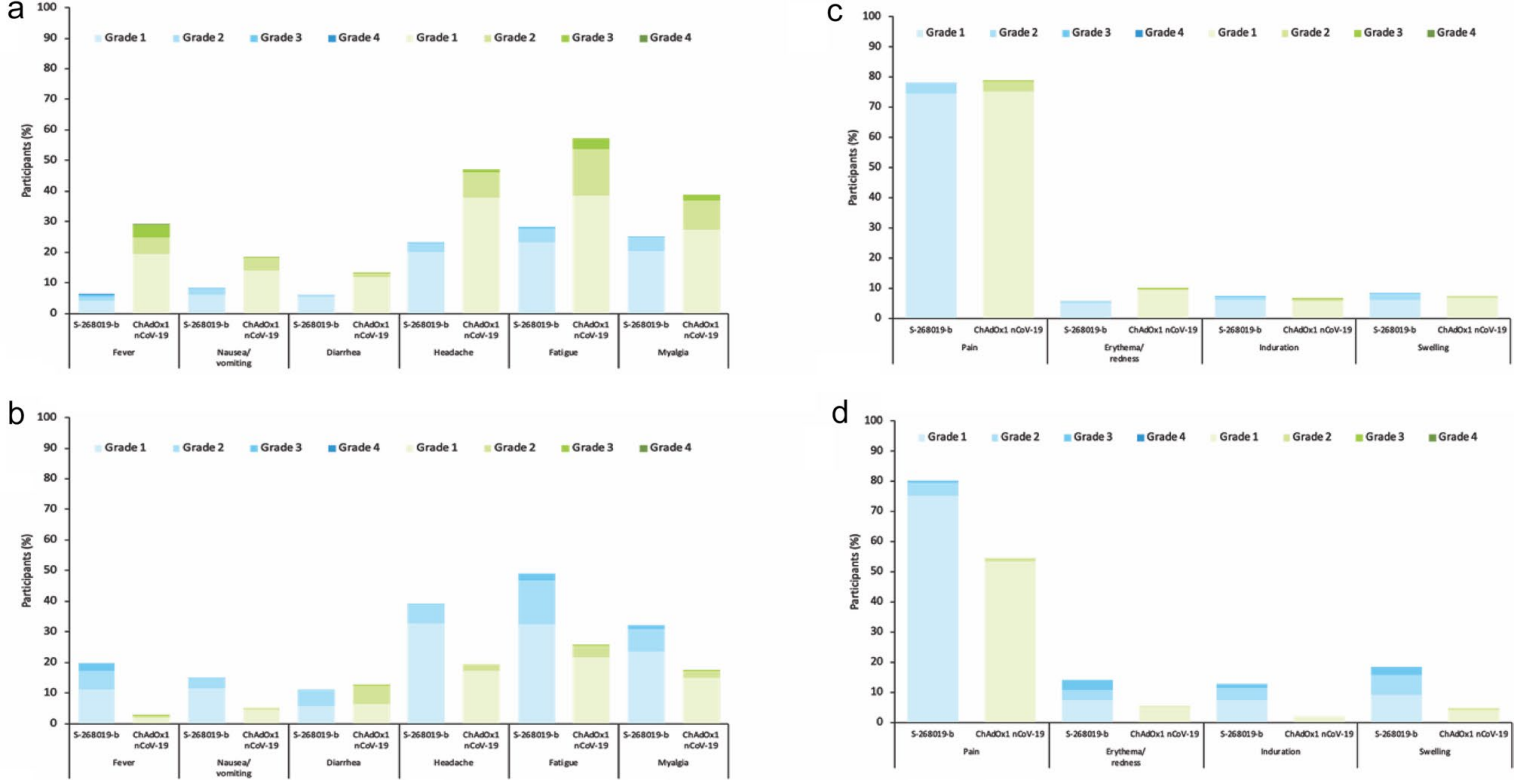
(**a**) Incidence of solicited systemic TRAEs by severity within 7 days after the first dose of the vaccine in the safety analysis set; (**b**) Incidence of solicited systemic TRAEs by severity within 7 days after the second dose of the vaccine in the safety analysis set; (**c**) Incidence of solicited local TRAEs by severity within 7 days after the first dose of the vaccine in the safety analysis set; (**d**) Incidence of solicited local TRAEs by severity within 7 days after the second dose of the vaccine in the safety analysis set. TRAE, treatment-related adverse event.

S-268019-b was well-tolerated without any new safety concerns; no treatment-related TRAEs were noted (Supplementary Table 1). Only one participant in each group reported AEs leading to discontinuation of study interventions; no deaths were reported till the data cut-off. Solicited AEs were mild to moderate in severity in most participants. The incidence of solicited systemic TRAEs within 7 days after both vaccinations in the safety analysis set are presented in Fig. 3a, b**,** whereas the corresponding solicited local TRAEs are presented in Fig. 3c, d. The incidence of solicited systemic TRAEs was higher after the first dose than the second dose in the ChAdOx1 nCoV-19 group, but higher after the second dose than the first dose in the S-268019-b group (Figs. 3a, b). Regardless, headache, fatigue, and myalgia were the most commonly reported solicited systemic TRAEs, while pain at the injection site was the most frequently reported solicited local TRAE. Overall, 1.3% and 7.9% of participants in the S-268019-b and ChAdOx1 nCoV-19 groups, respectively, experienced grade 3 solicited systemic TRAEs, and 0.3% of participants in both groups experienced grade 3 solicited local TRAEs. While 0.2% of participants in both groups experienced grade 4 solicited systemic TRAES, no grade 4 solicited local TRAEs were noted. There were no grade 5 systemic or local TRAEs within 7 days after both vaccinations in both S-268019-b and ChAdOx1 nCoV-19 groups.

Both S-268019-b and ChAdOx1 nCoV-19 elicited Th1-skewed responses evident in percentage of CD4 cells positive for IFN-γ and interleukin-2 (IL-2) in flow cytometry with intracellular cytokine staining (Supplementary Fig. 8a). The magnitude of effect at Day 43 was similar or greater with S-268019-b than with ChAdOx1 nCoV-19. Both vaccines had comparable IFN-γ spot forming units when assessed in the ELISPOT assay (Supplementary Fig. 8b).

**Figure 4:**
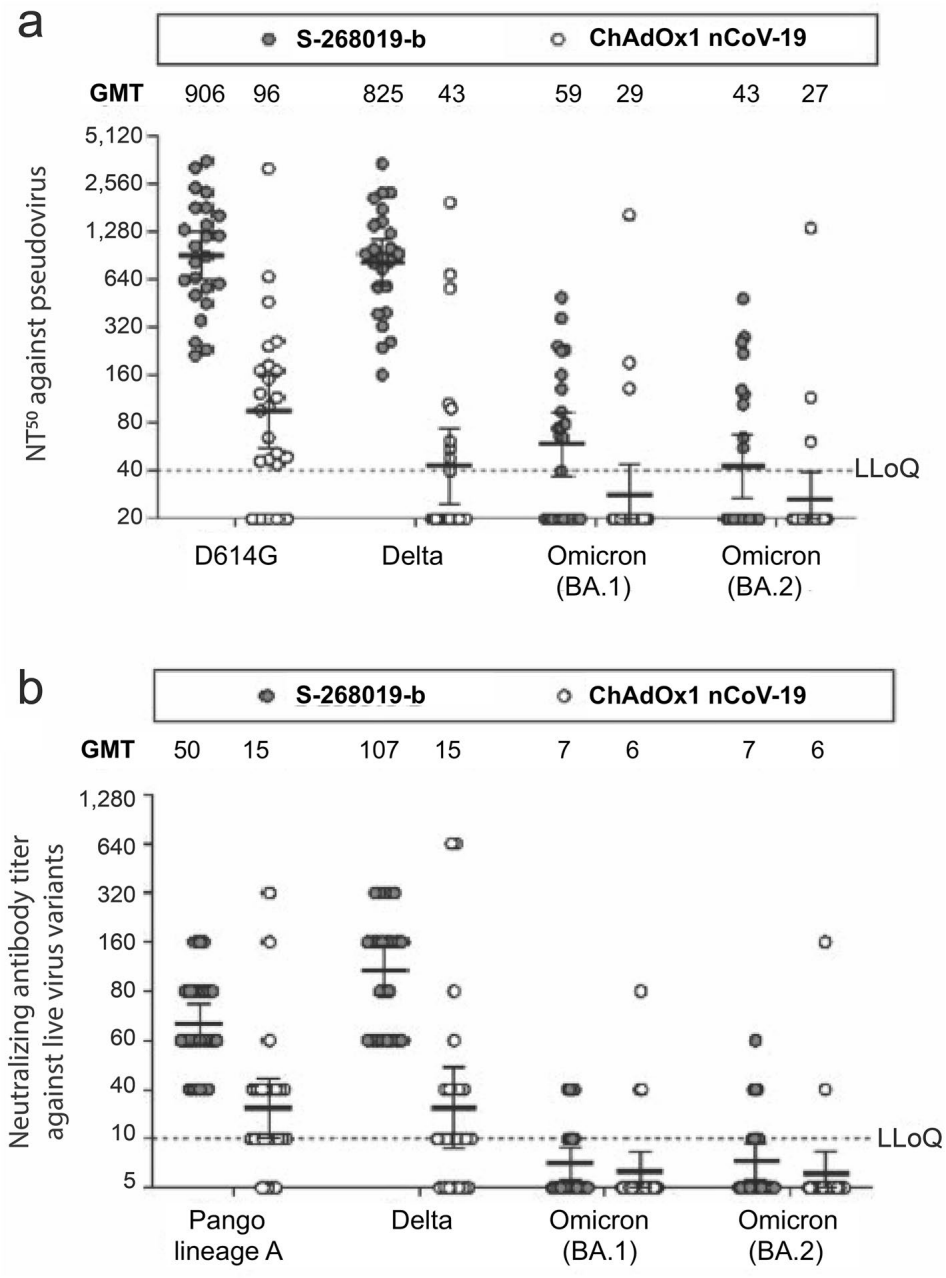
Neutralizing antibodies against the virus variants in the serum samples collected from vaccinated individuals at Day 57. Neutralizing antibody titers against (**a**) spike protein-bearing pseudoviruses and (**b**) live virus variants are shown. Selected samples from the immunogenicity subset (n = 24/group) were assessed for neutralizing antibody titer testing, and the sampling ensured no significant differences in age and neutralizing antibody titer against live wildtype virus on Day 57 compared with the entire cohort. The solid and open circles represent individual values for the S-268019-b and ChAdOx1 nCoV-19 groups, respectively. Titer values reported as below the LLoQ were replaced with 0.5 × LLoQ. Each bar represents the GMT with error bars indicating 95% CI. Samples were selected from the immunogenicity subset (n = 24/group) assessed on Day 57, and sampling ensured no significant differences in age and neutralizing antibody titer against live wildtype virus on Day 57 compared with the entire cohort. The 95% CIs were constructed using Student’s t distribution for log-transformed titers. CI, confidence interval; GMT, geometric mean titer; LLoQ, lower limit of quantification; NT50, serum titers at which 50% neutralization was achieved.

S-268019-b elicited about eightfold and 19-fold higher neutralizing antibodies against wildtype (D614G) and Delta pseudovirus variants, respectively, compared with ChAdOx1 nCoV-19 (Fig. 4a). However, compared with wildtype, there was a 15-fold and 21-fold reduction in GMT of the neutralizing antibodies elicited by S-268019-b against Omicron BA.1 and BA.2 variants, while GMTs in ChAdOx1 nCoV-19 were mostly below the detection limits. A similar trend was observed when tested with authentic SARS-CoV-2 variants (Fig. 4b).

## Discussion

In this randomized, controlled, phase 3 clinical trial, a superior neutralizing antibody response was shown 28 days after the second dose of the S-268019-b vaccine compared with the response to the ChAdOx1 nCoV-19 vaccine. A similar trend for higher magnitude of responses for neutralizing antibody and anti–SARS-CoV-2 S-protein IgG antibody titers in the S-268019-b group was observed in the follow-up period through Day 393.

S-268019-b showed good safety and immunogenicity in a preclinical study in cynomolgus monkeys^15^ and in a randomized, placebo-controlled, phase 1/2 study, a large open-label phase 2/3 study, and a randomized phase 2/3 study as a booster in Japanese participants^16,17^. In a preclinical study, immunization with 10 µg of S-910823 with A-910823 demonstrated protective efficacy against SARS-CoV-2 challenge according to genomic and subgenomic viral RNA transcript levels in nasopharyngeal, throat, and rectal swab specimens^15^. Furthermore, pathological analysis revealed no detectable vaccine-dependent enhancement of disease in the lungs of challenged vaccinated monkeys^15^. Additionally, based on the favorable results from the placebo-controlled phase 1/2 study and open-label phase 2/3 studies, S-268019-b is being evaluated in large phase 3 trials. The immunogenicity and safety results of S-268019-b from this large phase 3 trial are in alignment with previously published findings.

Some studies showed that neutralizing antibody titers correlate with vaccine efficacy in clinical trials of COVID-19 vaccines^10–12^. In our study, superior immunogenicity of S-268019-b when compared with a licensed active control vaccine with proven efficacy and effectiveness indicates the possibility that S-268019-b will provide good protection against symptomatic COVID-19 and SARS-CoV-2 infection. Clinical endpoints pertaining to the prevention of COVID-19 could not be analyzed in the current study as a result of limited statistical power, but another currently ongoing phase 3 study of S-268019-b will provide direct evidence on the potential of the vaccine to prevent COVID-19 (ClinicalTrials.gov number, NCT05212948). S-268019-b elicited robust and consistent immunogenicity in this study. The cytopathic assay in the present study showed lower levels of neutralizing antibodies in the ChAdOx1 nCoV-19 group compared with the published findings^18–20^. This likely reflects the differences in the cytopathic effect assays used across studies, and the one used in our study appears to have a lower sensitivity for detecting antibody at the lower end of the dynamic range, especially those elicited by ChAdOx1 nCoV-19. Such variability from using different assays was observed in the phase 1 trials of ChAdOx1 nCoV-19^21^. Indeed, most neutralizing antibody titer readings in the ChAdOx1 nCoV-19 group were below the LLoQ. However, seroconversion rates for the anti–SARS-CoV-2 S-protein IgG antibody 28 days after the second dose of S-268019-b vs ChAdOx1 nCoV-19 were 99.8% versus 98.2%, respectively. Moreover, a more sensitive cell viability assay confirmed our findings and showed a similar trend in favor of S-268019-b over ChAdOx1 nCoV-19 in neutralizing antibody responses. For primary endpoint comparison with this cell viability assay, approximately 60% of the neutralizing antibody titers for ChAdOx1 nCoV-19 were below the LLoQ. However, comparison of GMTs between S-268019-b and ChAdOx1 nCoV-19 groups using Wilcoxon rank-sum test, which is a non-parametric statistical hypothesis test, showed the robustness of the S-268019-b immunogenic response in both methods. The GMT of neutralizing antibody titer against wildtype (D614G) pseudovirus after two doses of ChAdOx1 nCoV-19 was 96 (Fig. 4a) and it was equivalent to 40 IU/mL as WHO international standard units^22^. Since GMTs of the neutralizing antibody titer against wildtype (D614G) pseudovirus in previous studies were 56.8 IU/mL^23^ and 39.0 IU/mL (median of inhibitory dilution (50%), 254 converted into WHO international standard unit)^12^, those were in the similar range observed in this study.

In the follow-up phase, the booster administration with S-268019-b elicited a durable neutralizing antibody response in both groups through the end of the study. Considering that the neutralizing antibodies tend to wane in 6–8 months following administration of the second dose for multiple vaccine types in previously published studies, booster application within that time frame could ensure maintenance of protective immunity^24,25^. Indeed, the fact that the neutralizing antibody titer following the booster dose was higher than that after the second dose suggests that immune system was adequately primed with two initial doses to mount the response in both treatment groups. Moreover, anti–SARS-CoV-2 S-protein IgG antibody titers were maintained through Day 393, further establishing the durability of the response. Notably, the magnitude of the neutralizing antibody and anti–SARS-CoV-2 S-protein IgG antibody responses as measured in GMTs was substantially higher in S-268019-b group versus that in ChAdOx1 nCoV-19, further corroborating the findings from the interim analysis 28 days after the second dose. It must be noted that results under controlled conditions in clinical trials may not always apply directly to the clinical settings.

Neutralizing antibody titers against the Omicron variant after two doses were lower for both S-268019-b and ChAdOx1 nCoV-19 compared with the wildtype with a pseudovirus assay, which aligns with previous reports on other vaccines, including ChAdOx1 nCoV-19^26,27^. Of note, studies have shown that a third dose of a COVID-19 vaccine increased vaccine effectiveness against the Omicron variant^28^. A third dose of BNT162b2 boosted neutralizing antibody titer against Omicron to a level just a couple of times lower than the neutralizing antibody titer against the wildtype, while neutralizing antibody titer against Omicron after just two doses was more than 10 times lower than that against the wildtype^29^. Moreover, in our earlier study, neutralizing antibody titers against the Omicron variants BA.1 and BA.2 elicited by a booster dose of S-268019-b in subjects who had received two doses of BNT162b2 as primary vaccination were similar to those seen with BNT162b2, which was reported to be effective against Omicron^17^. The booster dose of S-268019-b also elicited neutralizing antibody response against the more recent Omicron variants BA.4 and BA.5 in Japanese subjects who had received two doses of either mRNA-1273 or BNT162b2^30^.

Reactogenicity was mild to moderate in most participants and comparable between the two groups. No vaccine-related SAEs or AESIs were noted in the study. Headache, fatigue, and myalgia were the most common solicited systemic AEs, while pain at the injection site was the most frequently reported solicited local AE, all in agreement with previous published reports on S-268019-b and ChAdOx1 nCoV-19^16–19^. Solicited AEs were more frequent in the ChAdOx1 nCoV-19 group after the first dose, and in the S-268019-b group after the second dose, showing the same trend as reported previously with both vaccines^16,31^.

The S-268019-b formulation utilized a squalene-based adjuvant, which has been used in other vaccine formulations. A previous study has reported that an influenza vaccine containing a similar adjuvant, deployed in the 2009 pandemic, was associated with an increased risk of narcolepsy in a certain population^32^; however, the association has not been detected in other epidemiological studies. No neurological AESIs, including narcolepsy, were noted in the current study. As shown in preclinical studies, S-268019-b induced strong antibody responses, likely enhanced by the use of the adjuvant. In this study, S-268019-b, the adjuvanted vaccine, induced cytokine responses similar to ChAdOx1 nCoV-19, which produces competent levels of T-cell responses^33^. S-268019-b predominantly induced Th1 cell cytokines, while Th2 responses were minimal, aligning with the findings of a previous study^17^. Th1-skewed responses are considered important in avoiding the potential for risk related to vaccine-associated enhanced disease.

Our study has many strengths. A large phase 3 randomized clinical trial has high internal validity. Higher immunogenicity and tolerability of S-268019-b were demonstrated relative to an established active control, which is already approved for use globally. Favorable immunogenicity results at 28 days after the second dose were consistent across all age-groups assessed in the study, implying robustness of the results. Moreover, immunogenicity for both groups was maintained through the duration of the study, while the magnitude of the response was consistently and substantially higher in the S-268019-b group, establishing the durability and credibility of the initial response. The study also has its limitations, however. We presented information on safety from only the interim analysis (28 days following the second dose) as the most solicited adverse AEs occur within 7–10 days of vaccine administration and resolve within a few days^34^. We acknowledge that this may have precluded the possibility of capturing any long-term safety signals. However, participants were followed up, up to 1 year for additional immunogenicity endpoints, which provided valuable information on the durability of responses across vaccine platforms. The limited statistical power did not allow meaningful assessment of any clinical endpoints. Consequently, the study relied on surrogate data (immunogenicity in terms of neutralizing antibodies) rather than definite clinical endpoints (e.g., SARS-CoV-2 infection or time to first SARS-CoV-2 infection). Although the measurement of neutralizing antibodies may have been limited by the relatively low sensitivity in measuring the neutralizing antibody titer in the ChAdOx1 nCoV-19 group in one method, the more sensitive method also corroborated the same findings from the first method. Inclusion of a low number of non-Japanese study participants and a low number of elderly participants and those with risk factors for COVID-19 who need vaccination reduce generalizability of the current study. An ongoing study with S-268019-b specifically in the elderly is expected to address this gap. Regardless, our study adds value as a large comparative phase 3 study report of a recombinant protein vaccine showing superior neutralizing antibody induction than that observed with a globally COVID-19 vaccine in Japanese participants. The immune responses were durable and sustained through the duration of the study following booster administration. Importantly, the strong immunogenicity of the vaccine was not produced at the expense of tolerability. Future plans will include updating of the spike protein of S-268019-b in accordance with the recommendations of healthcare authorities in response to evolving SARS-CoV-2 variants, consistent with the examples of recombinant influenza vaccines.

## Conclusion

The results of this phase 3 study show superior neutralizing antibody responses 28 days after the second dose of and an acceptable safety profile for the S-268019-b vaccine with a modified recombinant spike protein of SARS-CoV-2 antigen, formulated with a squalene-based adjuvant, compared with the ChAdOx1 nCoV-19 vaccine.

### Supplementary Information

Supplementary Information 1.

Supplementary Figure 1.

Supplementary Figure 2.

Supplementary Figure 3.

Supplementary Figure 4.

Supplementary Figure 5.

Supplementary Figure 6.

Supplementary Figure 7.

Supplementary Figure 8.

## Data Availability

The datasets analyzed in this study are available on reasonable request of researchers through the clinical study data request platform (https://vivli.org/) after the researchers and SHIONOGI Group enter into a contract (which will have certain requirements with respect to personal data privacy, confidentiality, and compliance with the law). For further details, please refer to the websites of Shionogi & Co, Ltd (https://www.shionogi.com/shionogi/global/en/company/policies/shionogi-group-clinical-trial-data-transparency-policy.html).
