# Supplementary material for: A phase 3 randomized controlled trial of a COVID-19 recombinant vaccine S-268019-b versus ChAdOx1 nCoV-19 in Japanese adults
Source: Sci Rep. 2024 Apr 29;14:9830. doi: 10.1038/s41598-024-57308-3 (PMC11059267; doi:10.1038/s41598-024-57308-3)
Supplement: Supplementary file 1 — Supplementary Information 1. [file 41598_2024_57308_MOESM1_ESM.pdf]

Supplementary appendix

Anti-spike protein IgG titer measurement

Enzyme-linked immunosorbent assay (ELISA) was used to measure anti-spike protein IgG titers using duplicated samples. Full-length trimeric SARS-CoV-2 spike protein (BioServUK Ltd, BSV-COV-PR-35) was used as the immobilized antigen, and horseradish peroxidase conjugated goat-anti-human IgG (H+L) antibody (Invitrogen, A18811) was used as the detection antibody. Sample absorbance was measured as the difference between absorbance values at 405 nm and 490 nm, and the mean and coefficient of variation (CV) of absorbance for each duplicate were determined. The highest dilution factor with mean absorbance value more than or equal to the cutoff absorbance was considered the antibody titer for that sample.

Neutralizing antibody titer testing against ancestral SARS-CoV-2 by cytopathic effect (CPE) assay

Neutralizing antibody titers were measured with the SARS-CoV-2 JPN/TY/WK-521 ancestral strain (WK-521, Pango lineage A, kindly provided by the National Institute of Infectious Diseases, Japan) and transmembrane serine protease 2-expressing VeroE6 (VeroE6/TMPRSS2) cells. Heat-inactivated sera (56°C for approximately 30 minutes to remove non-specific inhibitors) were 2-fold serially diluted. Each sera sample was then mixed with 1:1 virus suspension containing 100 times the median tissue culture infectious dose ($TCID_{50}$)/well, and incubated for 1 hour at 37°C. The mixture of sera sample and virus was dispensed into each well of 96-well culture plates, and VeroE6/TMPRSS2 cell suspension (1×10^4 cells/well) was added to the wells. The plates were incubated at 37°C for 5 days with 5% CO_2, followed by examination for CPE under a microscope. Virus neutralization titer was defined as the reciprocal of the highest dilution resulting in equal to or more than 50% of CPE.

Neutralizing antibody titer testing against ancestral SARS-CoV-2 by cell viability assay

Neutralizing antibody titers were measured with the SARS-CoV-2, USA-WA1/2020 ancestral virus (Pango lineage A) and VeroE6 cells. The test sera were heat-inactivated to remove non-specific inhibitors at 56°C for 30 to 60 minutes prior to use in the assay, and 2-fold serially diluted. Each sera sample was mixed with 1:1 virus suspension, and incubated for 2 to 2.5 hours at 37°C. The mixture of sera sample and virus was transferred to each well on 96-well culture plates previously seeded with VeroE6 cells at 2×10^4 cells/well. The plates

were incubated at 37°C for 72 hours with 5% CO_2, followed by measurement for cell viability with CellTiter-Glo® Reagent (Promega) according to manufacturer's instructions. Neutralizing antibody titer was defined as the reciprocal of the highest dilution resulting in equal to or more than 50% cell viability.

Neutralizing antibody titer testing against live virus variants

Neutralizing antibody levels were assessed with the SARS-CoV-2 ancestral strain (Pango lineage A, 2019-nCoV/Japan/TY/WK-521/2020, GISAID ID: EPI_ISL_408667), Delta strain (hCoV-19/Japan/TY11-927-P1/2021, GISAID ID: EPI_ISL_2158617), Omicron BA.1 (hCoV-19/Japan/TY38-873/2021, GISAID ID: EPI_ISL_7418017) and BA.2 (hCoV-19/Japan/TY40-385/2022, GISAID ID: EPI_ISL_9595859), using the VeroE6/TMPRSS2 cells. Heat-inactivated sera samples (56°C for approximately 30 min to remove non-specific inhibitors) were 2-fold serially diluted. Each sera sample was then mixed with 1:1 live virus suspension containing either 100x, 100x, 10,000x and 10,000x $TCID_{50}$/well for WK-521, Delta, Omicron BA.1 and BA.2 strains, respectively, followed by incubation for 1 hour at room temperature. The mixture of sample and virus was dispensed (100 µL) into each well of culture plates in duplicates, and 100 µL of VeroE6/TMPRSS2 cell suspension (3×10^4 cells/well) was added to the wells. The plates were incubated at 37°C for 3 days with 5% CO_2, followed by measurement for cell viability with CellTiter-Glo® 2.0 Reagent (Promega) according to manufacturer's instructions. Neutralizing antibody titer was defined as the reciprocal of the highest dilution resulting in equal to or more than 50% cell viability.

Neutralizing antibody titer testing against pseudotyped virus variants

The stocks of D614G, Delta, Omicron BA.1, and BA.2 pseudotyped lentiviruses were diluted with the assay medium to prepare the viral suspensions of the same viral RNA copies/mL. Eleven serial 2-fold dilutions of heat-inactivated sera were mixed with an equal volume of viral suspension, followed by incubating for approximately 1 hour at 37°C for neutralization. After incubation, the mixture of sample and virus were added in duplicate to HEK293T stably expressing human ACE2 and TMPRSS2 cells, which were seeded into 96-well plates the day before the neutralization assay. Only virus suspension was added to virus control (VC) wells, which were placed on each plate. After incubating the plates at 37°C with 5% CO_2 for 2 days, cells were lysed and subjected to the luciferase assay to measure the Luciferase gene expression caused by lentiviral transduction. The intensity of luminescence

was measured by a microplate reader. Percent neutralization was calculated as the difference between relative light units (RLUs) of VC wells and test sample wells:

% Neutralization = 100% × [1 − (mean RLU of duplicate sample wells ÷ mean RLU of VC wells)]

The dilution factor achieving 50% of neutralization (50% neutralization titer; NT_{50}) was calculated by using the XLfit 5.3.1.3 software. When the percentage neutralization was less than 50% at the first dilution, the NT_{50} was expressed as the half of the first dilution factor. Geometric mean of the NT_{50} for each pseudovirus strain was calculated.

Intracellular cytokine staining by flow cytometry

Cytokine-producing T cells were identified by intracellular cytokine staining. Human peripheral blood mononuclear cells (PBMCs), thawed and rested for 4-5 hours in R10 supplemented medium, were restimulated (1.0×10^6 cells per well) with overlapping peptide pools of SARS-CoV-2 S (Miltenyi Biotec) and epitope peptide pools (Shionogi & Co., Ltd.) in the presence of Protein Transport Inhibitor Cocktail (Thermo Fisher Scientific K.K.) for 16 hours at 37°C. Controls were treated with a dimethyl sulfoxide-containing medium. Cells were stained for viability and surface markers (CD3 BV421 [BioLegend]; CD4 BV510 [BioLegend]; CD8 BB515 [BD Biosciences]) in staining buffer and Brilliant Stain Buffer Plus (BSB Plus, BD Horizon, according to the manufacturer's instructions) for 16-20 hours in a refrigerator. Next, the samples were fixed and permeabilized using the Cytofix/Cytoperm kit, according to the manufacturer's instructions (BD Biosciences). Intracellular staining for interferon gamma (IFN-γ) and interleukin (IL) (IFN-γ PE–Cy7 [BD Biosciences], IL-2 BB700 [BD Biosciences], IL-4 APC [BioLegend], and IL-5 PE [BioLegend]) was performed in the Perm/Wash buffer supplemented with BSB Plus (BD Horizon, according to the manufacturer's instructions) for 16-20 hours in a refrigerator. Samples were acquired on BD FACSCantoTM II (BD Biosciences) and analyzed with the FlowJo software version 7.6.5 (Becton, Dickinson and Company).

ELISPOT assay

IFN-γ ELISpot analysis was performed ex vivo using human PBMCs. Tests were performed in triplicate and with a positive control (PMA/ionomycin) and a negative control (medium), and the measurement was performed using the measurement kit, Human IFN-γ Single-Color ELISpot, white precoated (Cellular Technology Limited). CTL precoated plate was washed with D-PBS. Per well, 1.0×10^6 cells (for positive control: 5.0×10^4 cells) were stimulated for

19.5-20.5 hours with overlapping peptide pools of SARS-CoV-2 S (Miltenyi Biotec). Plates were scanned using ImmunoCapture 7.0.16.1 (Cellular Technology Limited) and counted using ImmunoSpot 7.0.30.4 (Cellular Technology Limited). Spot counts were displayed as mean values of each triplicate. The spot count values were normalized by the spot count value of negative control by using the following formula:

The spot count value = spot count value of peptide pools of SARS-CoV-2 S − spot count value of the negative control.

Supplementary Table 1: List of Institutional Review Boards (IRBs)

**Study Center Name**	**Name of IRB / IEC**
Clinical Research Hospital Tokyo	Review Board of Human Rights and Ethics for Clinical Studies Institutional Review Board
Shinei Diabetes Clinic	Review Board of Human Rights and Ethics for Clinical Studies Institutional Review Board
Yamazaki Otorhinolaryngology and Vertigo Clinic	Review Board of Human Rights and Ethics for Clinical Studies Institutional Review Board
Public Health Insurance Association Clinic	Review Board of Human Rights and Ethics for Clinical Studies Institutional Review Board
Shinagawa Strings Clinic	Review Board of Human Rights and Ethics for Clinical Studies Institutional Review Board
Irie Medical Clinic	Takahashi Pediatric Clinic Institutional Review Board
Aijinkai Takatsuki General Hospital	Takahashi Pediatric Clinic Institutional Review Board
Tokyo-Eki Center-Building Clinic	Tokyo-Eki Center-building Clinic Institutional Review Board
Fukuwa Clinic	Tokyo-Eki Center-building Clinic Institutional Review Board
Higashi Shinjuku Clinic	Institutional Review Board, P1 Clinic, Keiko-kai Medical Corporation
Samoncho Clinic	Yoyogi Mental Clinic Institutional Review Board
Tokyo Asbo Clinic	Review Board of Human Rights and Ethics for Clinical Studies Institutional Review Board
Motomachi Takatsuka Naika Clinic	Review Board of Human Rights and Ethics for Clinical Studies Institutional Review Board
Dojinkinenkai Meiwa Hospital	Review Board of Human Rights and Ethics for Clinical Studies Institutional Review Board
Shimamura Memorial Hospital	Review Board of Human Rights and Ethics for Clinical Studies Institutional Review Board
Koseikai Yotsubashi Clinic	Review Board of Human Rights and Ethics for Clinical Studies Institutional Review Board
Tenjin Sogo Clinic	Review Board of Human Rights and Ethics for Clinical Studies Institutional Review Board
Nihonbashi Sakura Clinic	Takahashi Pediatric Clinic Institutional Review Board
Sapporo Odori Endoscopy Clinic	Medical Corporation Cattleyakai Dr.Mano Medical Clinic Institutional Review Board
Sekino Hospital	Sekino Hospital Institutional Review Board

Supplementary Table 2: Key safety outcomes

Characteristic	S-268019-b	ChAdOx1 nCoV-19
	(N=611)	(N=610)
	n (%) [95% CI]; number of events	n (%) [95% CI]; number of events
**Participants with AEs**	572 (93.6) [91.4-95.4]; 3054	568 (93.1) [90.8-95.0]; 2840
**Death**	0 (0.0) [0.0-0.6];0	0 (0.0) [0.0-0.6]; 0
**Other SAEs**	6 (1.0) [0.4-2.1]; 6	1 (0.2) [0.0-0.9]; 1
**AEs of special interest**	0 (0.0) [0.0-0.6]; 0	0 (0.0) [0.0-0.6]; 0
**Medically attended AEs**	38 (6.2) [4.4-8.4]; 54	34 (5.6) [3.9-7.7]; 45
**AEs leading to discontinuation of study intervention**	1 (0.2) [0.0-0.9]; 1	1 (0.2) [0.0-0.9]; 2

AE, adverse event; CI, confidence interval; SAE, serious adverse event.
Potential immune-mediated diseases were collected as AEs of special interest.

Supplementary Figures

Supplementary Figure 1: Inclusion and exclusion criteria

COVID-19, coronavirus disease 2019; ICF, informed consent form; SARS-CoV-2, severe acute respiratory syndrome coronavirus 2.

Supplementary Figure 2: Study design and key assessments

AE, adverse event; COVID-19, coronavirus disease 2019; IgG, immunoglobulin G; NAb, neutralizing antibody; SAE, serious adverse event; SARS-CoV-2, severe acute respiratory syndrome coronavirus 2; Th1/Th2, T-helper 1/T-helper 2.
Regardless of the first and second vaccination at Day 1 and 29, participants who agreed to receive the third vaccination were given S-268019-b for the booster administration.

Supplementary Figure 3: GMTs for SARS-CoV-2 neutralizing antibody by visit in (a) the immunogenicity subset in the follow-up period (participants who received the third dose were excluded from the Day 393 analysis); (b) the immunogenicity subset following booster vaccination in the follow-up period (participants who received the third dose)

CI, confidence interval; GMT, geometric mean titer; NAb, neutralizing antibodies; SARS-CoV-2, severe acute respiratory syndrome coronavirus 2.
The groups represent the intervention at the time of primary vaccination.

Supplementary Figure 4: Seroconversion rate of S-268019-b for SARS-CoV-2 neutralizing antibody 28 days after the second doses of the respective vaccines when measured using the cytopathic effect assay

CI, confidence interval; SARS-CoV-2, severe acute respiratory syndrome coronavirus 2.

Supplementary Figure 5: Reverse cumulative distribution curves of neutralizing antibody titer in the ChAdOx1 nCoV-19 and S-268019-b groups when measured using the (a) cytopathic effect assay and the (b) cell viability assay

Supplementary Figure 6: GMT and GMTR of anti–SARS-CoV-2 S-protein IgG antibody titer 28 days after the second doses of the vaccines

CI, confidence interval; GMT, geometric mean titer; GMTR, geometric mean titer ratio; IgG, immunoglobulin G; LLoQ, lower limit of quantification; SARS-CoV-2, severe acute respiratory syndrome coronavirus 2.

Supplementary Figure 7: GMTs for Anti-SARS-CoV-2 S-protein (S1/S2) IgG antibodies by visit in (a) the immunogenicity subset in the follow-up period (participants who received the third dose were excluded from the Day 393 analysis); (b) the immunogenicity subset following booster vaccination in the follow-up period (participants who received the third dose)

CI, confidence interval; GMT, geometric mean titer; IgG, immunoglobulin G; SARS-CoV-2, severe acute respiratory syndrome coronavirus 2.
The groups represent the intervention at the time of primary vaccination.

Supplementary Figure 8: (a) Flow cytometry with intracellular cytokine staining and (b) ELISPOT assay
Percentage of CD4 cells are presented as mean (95% CI). Spot forming units are presented as mean (SD).

CI, confidence interval; IFN-γ, interferon gamma; IL, interleukin; SD, standard deviation; Th1/Th2, T-helper 1/T-helper 2.
