# Supplementary figures and images for: A phase 3 randomized controlled trial of a COVID-19 recombinant vaccine S-268019-b versus ChAdOx1 nCoV-19 in Japanese adults
Source: Sci Rep. 2024 Apr 29;14:9830. doi: 10.1038/s41598-024-57308-3 (PMC11059267; doi:10.1038/s41598-024-57308-3)

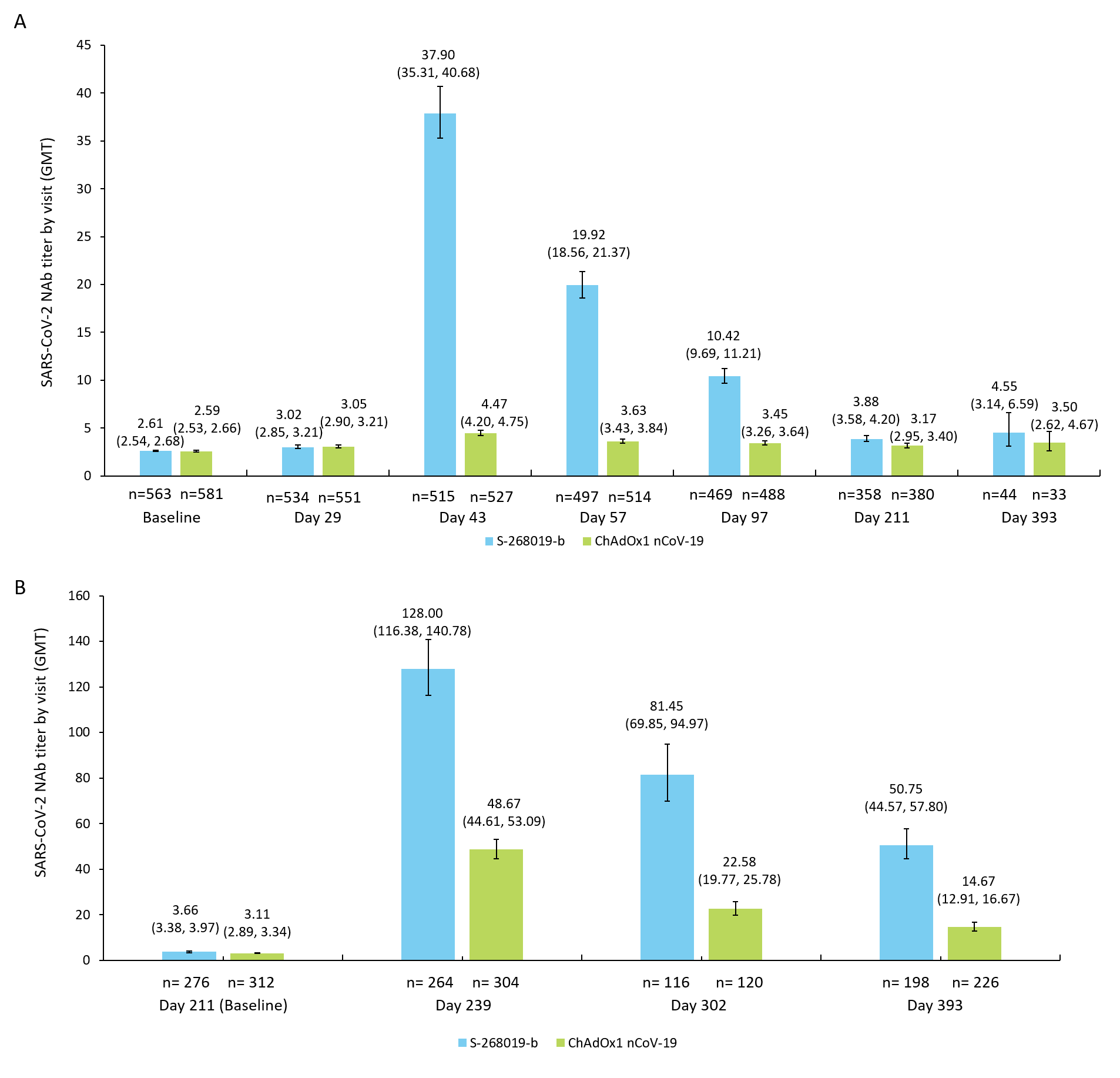

Supplement: Supplementary file 4 — Supplementary Figure 3. [file 41598_2024_57308_MOESM4_ESM.tif]

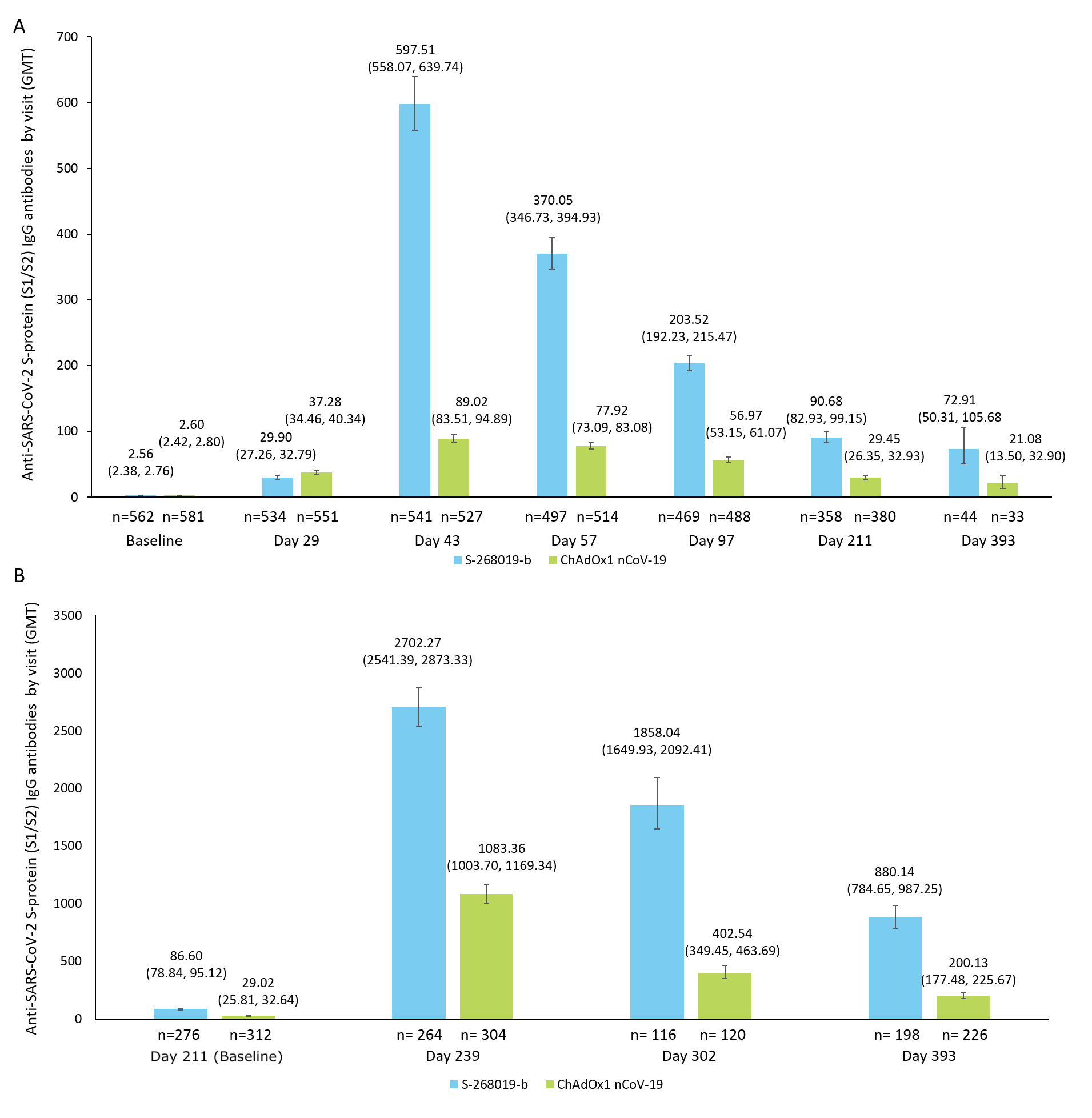

Supplement: Supplementary file 8 — Supplementary Figure 7. [file 41598_2024_57308_MOESM8_ESM.tif]

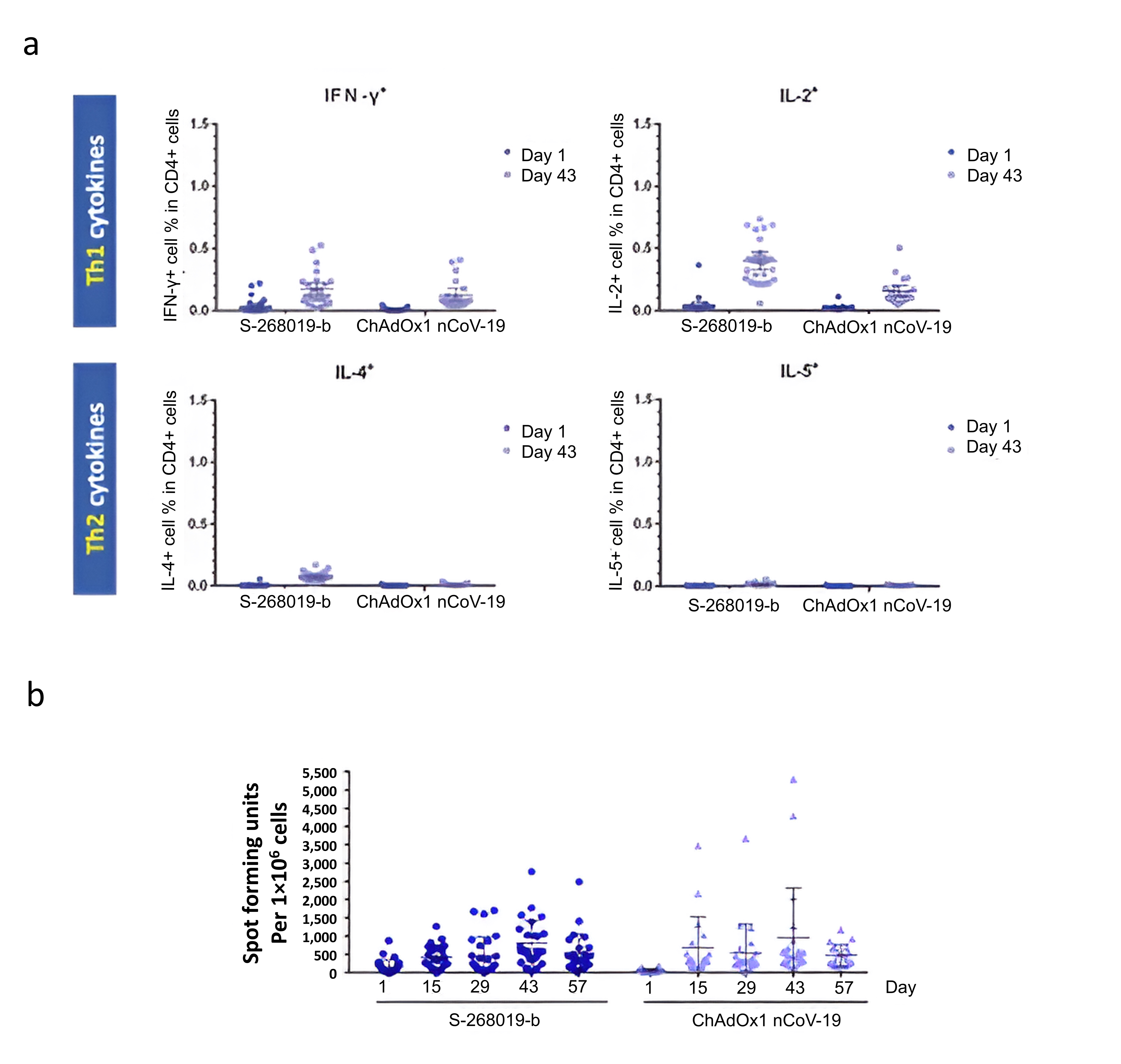

Supplement: Supplementary file 9 — Supplementary Figure 8. [file 41598_2024_57308_MOESM9_ESM.jpg]
